# Systematic review of the clinical manifestations of glucose-6-phosphate dehydrogenase deficiency in the Greater Mekong Subregion: implications for malaria elimination and beyond

**DOI:** 10.1136/bmjgh-2017-000415

**Published:** 2017-08-19

**Authors:** Ken Ing Cherng Ong, Hodaka Kosugi, Sophea Thoeun, Hitomi Araki, Moe Moe Thandar, Moritoshi Iwagami, Bouasy Hongvanthong, Paul T Brey, Shigeyuki Kano, Masamine Jimba

**Affiliations:** 1 Department of Community and Global Health, Graduate School of Medicine, The University of Tokyo, Tokyo, Japan; 2 SATREPS Project (JICA/AMED) for Parasitic Diseases, Vientiane Capital, Lao People's Democratic Republic; 3 Institut Pasteur du Laos, Ministry of Health, Vientiane Capital, Lao People's Democratic Republic; 4 Department of Tropical Medicine and Malaria, Research Institute, National Center for Global Health and Medicine, Tokyo, Japan; 5 Center of Malariology, Parasitology and Entomology, Ministry of Health, Vientiane Capital, Lao People's Democratic Republic

**Keywords:** malaria, greater mekong subregion, G6PD deficiency

## Abstract

**Introduction:**

To achieve malaria elimination in the Greater Mekong Subregion (GMS) by 2030, proper case management is necessary. 8-aminoquinolines, such as primaquine, are the only available medicines effective in preventing relapse of the hypnozoite stage of *Plasmodium vivax*, as well as the onward transmission of *Plasmodium falciparum*. However, primaquine can cause haemolysis in individuals who have glucose-6-phosphate dehydrogenase deficiency (G6PDd). We conducted a systematic review on the reported clinical manifestations of G6PDd to provide a comprehensive overview of the situation in the GMS.

**Methods:**

The protocol for this systematic review was registered on PROSPERO: International prospective register of systematic reviews (CRD42016043146). We searched the PubMed/MEDLINE, CINAHL, and Web of Science databases for published articles describing the clinical manifestations of G6PDd in the GMS. We included articles of all study designs from inception until 31 July 2016, reporting the clinical manifestations of G6PDd. We then performed a narrative synthesis of these articles.

**Results:**

We included 56 articles in this review, 45 of which were from Thailand. Haemolysis in G6PD-deficient individuals was caused not only by primaquine but also by other medicines and infections. Other clinical manifestations of G6PDd that were found were favism, neonatal jaundice and chronic non-spherocytic haemolytic anaemia. G6PDd also influenced the clinical presentations of genetic disorders and infections, such as thalassemia and typhoid fever.

**Conclusion:**

As G6PDd also affects the clinical presentations of other infections, the benefits of G6PD testing and proper record keeping transcend those of malaria case management. Therefore, healthcare workers at the community level should be made familiar with complications resulting from G6PDd as these complications extend beyond the scope of malaria.

**Key questions**

**What is already known about this topic?**

- Primaquine plays an important role in malaria elimination.
- Primaquine is contraindicated in individuals with glucose-6-phosphate dehydrogenase (G6PD) deficiency.
- Testing for G6PD deficiency has the potential to help healthcare workers provide proper treatment to patients with malaria who are G6PD-deficient.

**What are the new findings?**

- Testing for G6PD deficiency and proper record keeping are effective in helping healthcare workers make informed decisions about providing treatment to patients with malaria.
- G6PD deficiency also affects the clinical presentation of other infections such as dengue fever and typhoid fever; therefore, the benefits of G6PD testing and proper record keeping go beyond the management of malaria.

**Recommendations for policy**

- To avoid jeopardising the lives of G6PD-deficient individuals, their G6PD status should be determined before primaquine prescription.
- Systematic G6PD screening and proper record keeping of the most at risk populations are recommended.
- Training healthcare workers and equipping local facilities to enable better management of complications due to G6PD deficiency are recommended.

## Introduction

Glucose-6-phosphate dehydrogenase (G6PD) deficiency is the most common enzyme deficiency in the world, affecting over 400 million people.^1^ Most people with G6PD deficiency (G6PDd), however, do not exhibit symptoms unless exposed to oxidative stress.^1 2^ Oxidative stress can be triggered by medicines, infections, fava bean consumption, or even strenuous physical exercise.^1^ The main clinical manifestations of G6PDd are acute haemolytic anaemia, chronic non-spherocytic haemolytic anaemia (CNSHA), neonatal jaundice and favism.^1 2^

The WHO classifies G6PDd into five different categories according to the severity of enzyme deficiency.^1 3^ A Class I deficiency is defined as severe deficiency and is associated with CNSHA. A Class II deficiency is also defined as severe deficiency and the enzyme activity is 1%–10% of normal activity. Individuals with a Class III deficiency are moderately deficient and their enzyme activity is 10%–60% of normal activity. Class IV and Class V individuals have normal and increased activity with an enzyme activity of 60%–150% and over 150%, respectively.^1 3^

The two most common G6PDd variants are the G6PD A- and G6PD Mediterranean variants.^4^ G6PD A- is common across the African continent and is categorised as a Class III deficiency, whereas G6PD Mediterranean is common among Italians, Arabs, and Jews, and is categorised as a Class II deficiency.^4^ In Southeast Asia, the most common variant in Myanmar and Thailand is G6PD Mahidol (Class III), whereas in Laos and Cambodia, the most common variant is G6PD Viangchan (Class II).^5 6^

Acute haemolysis can occur in a G6PD-deficient person who is exposed to antimalarials from the 8-aminoquinoline family.^7^ However, 8-aminoquinoline antimalarials, such as primaquine, remain the only medicines available against chronic infection and relapse caused by *Plasmodium vivax* and *Plasmodium ovale*, and the onward transmission of *Plasmodium falciparum*.^7^

The Greater Mekong Subregion (GMS) comprises Lao People's Democratic Republic (PDR), Thailand, Cambodia, Vietnam, Myanmar and Yunnan Province and Guangxi Zhuang Autonomous Region of the People's Republic of China. The GMS encompasses an area of 2.6 million km^2^ and sustains over 326 million people.^8^ It is characterised by rapid economic development, high population densities, diverse geographical environments and the mass movement of people across borders.^9 10^ Despite current progress, malaria elimination efforts in GMS countries have been hampered by the emergence and spread of the artemisinin resistant *P. falciparum* malaria parasite.^11^ Moreover, *P. vivax* is extremely resilient to elimination due to the dormant hypnozoite stage which occurs in the liver and the parasite's ability to spread via mosquitoes before the onset of clinical symptoms. Therefore, proper utilisation of an antihypnozoite such as primaquine to eliminate malaria is warranted.^12^

G6PDd variants are diverse and each deficient individual can respond differently when exposed to oxidative stress caused by primaquine.^13^ In rural endemic areas where knowledge is often limited, G6PD-deficient individuals might not associate the onset of symptoms such as weakness, jaundice and shortness of breath with the administration of primaquine.^14^ Therefore, determining the G6PD status of the patient before starting any primaquine regimen is crucial, particularly in such settings. Moreover, it is important to understand the major clinical manifestations and burden of G6PDd in a region before implementing a drug administration regimen, so that people are not exposed to unnecessary risks.

Beyond malaria, G6PDd is also relevant in other contexts.^1^ For example, patients with viral hepatitis with G6PDd are at risk of serious complications resulting from acute renal failure.^1^ Moreover, neonates with G6PDd are at a higher risk of neonatal hyperbilirubinaemia which can result in irreversible neurological damage, a condition known as kernicterus.^1 4^ Although the WHO recommends that all newborns be screened for G6PDd in populations where more than 3%–5% of males are affected,^3^ this is not performed in many GMS countries due to financial constraints.^15^

Despite the high burden of G6PDd in malaria endemic GMS, there remains a lack of data on the major clinical complications of G6PDd. Therefore, we conducted this review to identify the major clinical manifestations and complications of G6PDd in GMS countries.

## Methods

### Search strategy

We prepared a protocol and registered it on PROSPERO: International prospective register of systematic reviews (CRD42016043146), ((online supplementary file 1), Preferred Reporting Items for Systematic Reviews and Meta-Analyses (PRISMA) flow diagram; (online supplementary file 2), study protocol; (online supplementary file 3), PRISMA checklist). We searched PubMed/MEDLINE, CINAHL, and Web of Science for published studies in the English language from inception until 31 July 2016. Our search strategy combined both Medical Subject Heading terms and relevant keywords. We used the following combinations:

10.1136/bmjgh-2017-000415.supp1 Supplementary file 1

10.1136/bmjgh-2017-000415.supp2 Supplementary file 2

10.1136/bmjgh-2017-000415.supp3 Supplementary file 3

(glucose-6-phosphate dehydrogenase deficiency OR G6PDd OR glucosephosphate dehydrogenase deficiency OR G-6-PD deficiency OR G6PD deficiency OR glucose-6-phosphate dehydrogenase OR G6PD OR G-6-PD) AND (Greater Mekong Subregion OR GMS OR Lao People's Democratic Republic OR Lao PDR OR Laos OR Thailand OR Vietnam OR Viet Nam OR Cambodia OR Myanmar OR Burma OR People's Republic of China OR Guangxi Zhuang Autonomous Region OR Yunnan Province OR Yunnan OR PRC).

Tasks were divided equally among the five reviewers (KICO, HK, ST, HA and MMT) for each stage of the review process, which included searching, screening and data extraction. We resolved all disagreements and conflicts through group meetings and discussions with the review team members.

### Inclusion and exclusion criteria

We included original research of all study designs, including clinical trials, cohort studies, case–control studies, cross-sectional studies, case series and case reports according to the following criteria: G6PDd was the target condition, G6PDd was identified after the manifestation of clinical symptoms, clinical information was available and the study population was from the GMS. We excluded review papers and studies on migrant populations from outside the GMS residing within the GMS.

### Data extraction and data synthesis

We used a standardised data extraction form (online supplementary table—Summary of included studies) to summarise relevant information from included studies. We retrieved the following information from each study: study design, location, year of publication, population, diagnostic methods, exposures and clinical outcomes.

10.1136/bmjgh-2017-000415.supp4 Supplementary file 4

Subsequently, we conducted a narrative synthesis of all the included studies.

## Results

We found 839 articles through the database searches: 728 articles from PubMed, four articles from CINAHL and 107 articles from Web of Science. After removing 94 duplicates, we screened the abstracts of 745 articles. We excluded a further 672 articles during abstract screening. Articles were excluded when there was no clinical information present, G6PDd was not the target condition or the study was a review or an opinion article. We then screened the full text of 73 articles and excluded 19 articles for the reasons mentioned above. We also included two articles found in a report from the WHO.^16^ In total, we included 56 articles in our qualitative analysis (online supplementary file 1—PRISMA flow diagram).

Forty-five of the 56 articles were from Thailand, four were from Cambodia, two were from Lao PDR, three were from Myanmar and two were from Vietnam. Forty-seven studies were observational (case–control, cohort, case report, case series or cross-sectional) and nine were experimental (randomised controlled trial or quasi-experimental studies) (table 1). In general, both the study design and the sample size of the included articles were heterogeneous.

**Table 1:** Summary of included articles by country, major clinical symptoms and type of study

Country	Major clinical symptoms	Type of study						
		Case–control	Cohort	Case report	Cross-sectional	Case series	Experimental	Total
Cambodia	Primaquine-induced haemolysis, anaemia in glucose-6-phosphate dehydrogenase deficiency individuals during malaria infection	0	1	0	1	1	1	4
Lao PDR	Haemoglobinuria, haemolytic anaemia	0	0	2	0	0	0	2
Myanmar	Acute bilirubin encephalopathy	0	1	0	1	0	1	3
Thailand	Haemoglobinuria, haemolysis, jaundice, chronic non-spherocytic haemolytic anaemia, favism	1	6	8	15	8	7	45
Vietnam	Haemoglobinuria	0	0	0	2	0	0	2
Total		1	8	10	19	9	9	56

### Clinical manifestations of G6PDd in the GMS

#### Haemolysis

We found a total of seven articles on primaquine-induced haemolysis.^17–23^ Of these, five were from Thailand and two were from Cambodia. In three studies, a 15 mg daily dose of primaquine for 14 days was given to patients.^18 20 21^ The degree of haemolysis reported in the three studies was moderate and self-limiting.

In a study in Cambodia on the tolerability of a 0.75 mg/kg of primaquine weekly regimen, a male patient with G6PDd developed clinically significant anaemia (a haemoglobin concentration of 7.2 g/dL) which required a transfusion.^23^ However, the patient took cimetidine and ciprofloxacin before enrolling in the study and this could have contributed to his deteriorating condition.^23^

A single dose of 45 mg of primaquine was given to seven healthy men in Thailand.^17^ However, the degree of haemolysis reported in this study was mild. In another study, a Thai soldier presented with vomiting and dark urine after taking 15 mg of primaquine daily for 3 days. His minimum haematocrit value was 19.0%. His condition worsened after a few days and he was also diagnosed with acute renal failure. Later, he was found to have G6PDd.^22^

A primaquine overdose case was reported on the Thai-Myanmar border.^19^ The patient was given primaquine and chloroquine for a *P. vivax* infection. However, the patient was not tested for G6PDd and the instructions were not given in a language understood by the patient.^19^ This resulted in the patient taking 11 primaquine tablets (15 mg base per tablet) in one sitting. His haemoglobin concentration was 6.9 g/dL. Nevertheless, his condition improved following blood transfusions. No deaths were reported in any of the studies.

A total of nine articles mentioned infection-induced haemolysis.^24–32^ The types of infections reported were typhoid fever,^24 29 31^ dengue infection,^25 27 28^ falciparum malaria^26^ and viral hepatitis.^30^ One of the major complications described among patients with typhoid fever or dengue infection who also had G6PDd was acute haemolysis. Patients with viral hepatitis suffered from acute haemolysis (haemoglobin concentration as low as 6.0 g/dL in one patient), as well as severe hyperbilirubinaemia. In the article on falciparum malaria infection, no significant difference in the haemoglobin concentration was observed between G6PD normal and G6PD-deficient patients. No deaths were reported in any of these studies.

Four articles, three from Thailand and one from Lao PDR mentioned drug-induced haemolysis in individuals with G6PDd.^32–35^ The article from Lao PDR described the symptoms of soldiers who had taken a single high dose of chloroquine (600 mg) as malaria prophylaxis.^35^ They experienced severe haemolytic anaemia, dark urine and acute renal failure.^35^ In one study from Thailand, among patients with G6PDd, the causes of acute haemolysis, dark urine or jaundice were not characterised. However, medicines such as aspirin and phenacetin were implicated.^33^ No deaths were reported in these studies.

### Favism, neonatal jaundice and CNSHA

We found two articles from Thailand that mentioned favism.^36 37^ The first study was conducted on 225 (210 boys and 15 girls) children with G6PDd.^36^ Among 225 children, eight children (3.6%), all of whom were boys, were found to have favism. These children presented with sudden onset of anaemia within 1–3 days after consumption of dried fava beans. Five out of eight children required blood transfusions. No deaths were reported. In the second study, the authors reported no association between fava bean consumption and the onset of haemolysis among individuals with G6PDd.^37^

A total of four articles examined neonatal jaundice, all of which were from Thailand.^32 38–40^ Two studies were conducted in the northern Thai city of Chiang Mai. One study described two cases of moderate to severe neonatal jaundice among 35 infants with G6PDd.^38^ The other study from Chiang Mai included two series. The first series reported 25 cases of severely jaundiced infants, of which 16 had G6PDd. The second series described 17 infants with G6PDd, of which five had severe jaundice.^39^ Of these 21 severely jaundiced infants with G6PDd, 11 were female. In another study from southern Thailand, neonatal jaundice was detected in 85% of 225 patients with G6PDd.^32^ A study from Bangkok reported a higher percentage of jaundice among male infants with G6PDd (49.2%) compared with male infants without G6PDd (23.7%).^40^

CNSHA was mentioned in two articles from Thailand.^28 41^ The first article described the case of an 11-year-old boy who was anaemic and required a blood transfusion at the age of 5 months.^41^ The boy was found to be carrying a mutation unlike any previously reported variants, with the variant subsequently named G6PD Bangkok.

The second article provided details of two families affected by CNSHA, including the family of the previously mentioned boy with G6PD Bangkok.^28^ The boy with G6PD Bangkok was followed until he was 53 years old. He had two daughters, both of whom had neonatal jaundice. In the second family, two male siblings suffered from neonatal jaundice as well as several episodes of haemoglobinuria following febrile illnesses during childhood. They were found to be the carriers of a novel mutation that was subsequently named G6PD Bangkok Noi.

### G6PDd and genetic disorders, infections or haematological conditions

Seven articles described the dual burden of G6PDd and other infections or genetic disorders.^42–48^ Five articles discussed G6PDd and malaria, three of which were from Thailand, one was from Cambodia and one was from Myanmar.^42 43 45–47^ In the article from Myanmar, the double red cell genetic disorders of thalassemia and severe G6PDd were reported to confer a certain degree of protection against falciparum malaria.^47^ Two articles from Thailand found that having G6PDd was associated with having typhoid fever and hereditary elliptocytosis, respectively.^44 48^

Eight articles examined the relationship between G6PDd and other haematological conditions.^49–56^ In a study from Myanmar, G6PDd was found to be a risk factor for acute bilirubin encephalopathy among neonates.^49^ A significant increase in mean corpuscular reticulocyte volume was reported among patients with anaemia with G6PDd in a study from Thailand.^50^ Another Thai study found that neonatal hyperbilirubinaemia with bilirubin encephalopathy was the major deleterious effect of G6PDd.^51^ Two studies from Vietnam reported an association between having G6PDd and developing blackwater fever syndrome.^52 56^ In a study on neonates in Thailand, having G6PDd was found to be associated with developing neonatal hyperbilirubinaemia.^54^ G6PDd was not reported to have any additional adverse effects on the haematological parameters of homozygous haemoglobin E individuals in Thailand.^55^

### Other findings

A total of 11 articles discussed the effects of several antimalarial medicines on patients with G6PDd.^57–67^ Nine articles were from Thailand, one was from Cambodia and one was from Myanmar. The medicines examined were primaquine, sulfalene–trimethoprim, sulformethoxine–pyrimethamine, mefloquine, elubaquine, quinine and doxycycline. No severe adverse events were reported in patients with G6PDd.

One article from Thailand reported that highly active antiretroviral therapy did not cause haemolytic anaemia nor did it cause hyperbilirubinaemia in G6PD-deficient patients living with HIV.^68^

Other findings on the clinical manifestations of G6PDd were described in four articles.^69–72^ A study in Thailand found that among females with the G6PD Viangchan mutation, strenuous exercise induced a significantly higher increase in total microparticle level (an indicator of oxidative damage) compared with age-matched G6PD normal females.^69^ In a study from Thailand, the authors suggested that excessive fluoride in drinking water might worsen the effect of iron deficiency anaemia, thalassemia and G6PDd.^71^ In another study from Thailand, among 165 neonates, G6PDd was the main reason 22 neonates (13.4%) required exchange transfusion therapy. In contrast, G6PDd together with ABO incompatibility was the main reason 11 neonates (6.7%) required exchange transfusion therapy.^72^

## Discussion

This systematic review has three major findings. First, the major clinical manifestation of G6PDd in the GMS was haemolysis induced by not only primaquine but also other medicines and infections. Second, favism, neonatal jaundice and CNSHA were reported as clinical manifestations of G6PDd. Finally, G6PDd affected the clinical presentations of genetic disorders and infections, such as thalassemia and typhoid fever.

In two case reports from Thailand, two male patients were prescribed primaquine without G6PD testing.^19 22^ Their conditions worsened after a few days, and both required blood transfusions; following transfusions, both patients recovered. In the GMS, many malaria endemic villages are remote, difficult to access and have very weak health infrastructures.^10^ Therefore, when haemolysis occurs in such settings, the local health facilities are unlikely to be able to provide appropriate medical care, potentially resulting in the loss of life. G6PD testing is particularly crucial under such conditions.

Knowing the G6PD status of patients might be beneficial not only in the context of malaria but also in the context of other infections such as typhoid fever, dengue infection and viral hepatitis. Haemolysis can also occur in G6PD-deficient patients who are coinfected with the abovementioned infections.^24 25 27–31^ As typhoid fever and dengue infection are prevalent in the GMS,^73 74^ knowing the G6PD status of local populations might help healthcare workers improve management of these infections in people with G6PDd.

Although favism is a primary manifestation of G6PDd,^1^ we found only two studies that discussed favism in the GMS, both of which were from Thailand.^36 37^ Favism, which results from the ingestion of fava beans, usually manifests as acute haemolytic anaemia and severe haemoglobinuria.^1^ In GMS countries such as Thailand, fava beans are usually consumed as a snack and not as a staple food.^37^ Moreover, as favism is more common among children than adults, this might explain why the study on adults found no association between haemolysis and fava bean consumption among individuals with G6PDd.^37^ In contrast, the incidence of favism was 3.6% in the study on children with G6PDd.^36^ However, the study designs of the two studies must also be taken into account. In the first study, only children with G6PDd were included.^36^ In the second study, hospital patients aged 16–60 were included and their G6PD status was determined later.^37^ Therefore, this could have biased the results and explained the higher number of favism cases in the study on children. In addition, favism could also be under-reported in the GMS.

The lack of information on neonatal jaundice and G6PDd from GMS countries other than Thailand indicates that neonatal jaundice might be under-reported. Neonatal jaundice, which can lead to permanent brain damage in a condition called kernicterus, is one of the most devastating consequences of G6PDd.^75^ In addition, neonates with G6PDd are more likely to develop neonatal jaundice compared with neonates with normal G6PD activity.^2^ Knowing the G6PD status of individuals, especially in the GMS, can also help with better case management of neonatal jaundice in neonates with G6PDd.

Even though CNSHA is a rare condition among people with G6PDd, the lack of information from other GMS countries besides Thailand might be due to under-reporting. The two variants described in the two studies were G6PD Bangkok and G6PD Bangkok Noi. The most serious form of infection-induced haemolysis was reported to be due to dengue haemorrhagic fever, which required blood transfusions.^28 41^ As dengue infection is prevalent in the GMS^74^ and CNSHA might be under-reported, the benefits of G6PD screening might also be applicable to G6PD-deficient individuals with CNSHA.

Treatment of complications resulting from G6PDd, such as blood transfusions, can be costly and require proper facilities and trained personnel. A cost study in Brazil estimated that the average cost of treatment of severe adverse events due to primaquine among patients with G6PDd is about US$4.8 million per year.^76^ Supplying G6PD test kits could help prevent severe adverse events due to primaquine. However, this alone may not be a financially sustainable solution as G6PDd also affects the clinical manifestations of other diseases and infections. Our results indicate that appropriate management by trained healthcare workers also has the potential to prevent the loss of life due to haemolysis.

Nevertheless, the evidence from this review should be interpreted with caution as we have only included papers published in the English language. Moreover, most studies we found were from Thailand. Consequently, the evidence here might not be exhaustive of all the clinical manifestations of G6PDd in the GMS. However, based on the predicted prevalence and predicted risk of haemolysis of the G6PDd variants in the GMS,^77^ the clinical manifestations of G6PDd in the GMS have most likely been under-reported. In addition, the clinical manifestations described in our systematic review are consistent with those reported in a systematic review of the clinical manifestations of G6PDd in Latin America.^78^

## Conclusion

Prescription of primaquine should be preceded by prior assessment of a patient's G6PD status. Moreover, systematic screening and record keeping of G6PDd would be beneficial and cost-effective in the GMS. When trained healthcare workers properly manage severe adverse events, they can prevent the loss of life due to haemolysis in patients with G6PDd. Healthcare workers at the community level should be made familiar with symptoms and complications due to G6PDd and trained to take appropriate action such as referring a patient to a better equipped health facility. Finally, G6PD status is important not only in the context of malaria but also in the context of other infections such as typhoid fever and dengue fever, which are prevalent in the GMS.
