# Supplementary material for: Systematic review of the clinical manifestations of glucose-6-phosphate dehydrogenase deficiency in the Greater Mekong Subregion: implications for malaria elimination and beyond
Source: BMJ Glob Health. 2017 Aug 19;2(3):e000415. doi: 10.1136/bmjgh-2017-000415 (PMC5656182; doi:10.1136/bmjgh-2017-000415)
Supplement: Supplementary file 1 [file bmjgh-2017-000415supp001.pdf]

S1 File. PRISMA flow diagram.

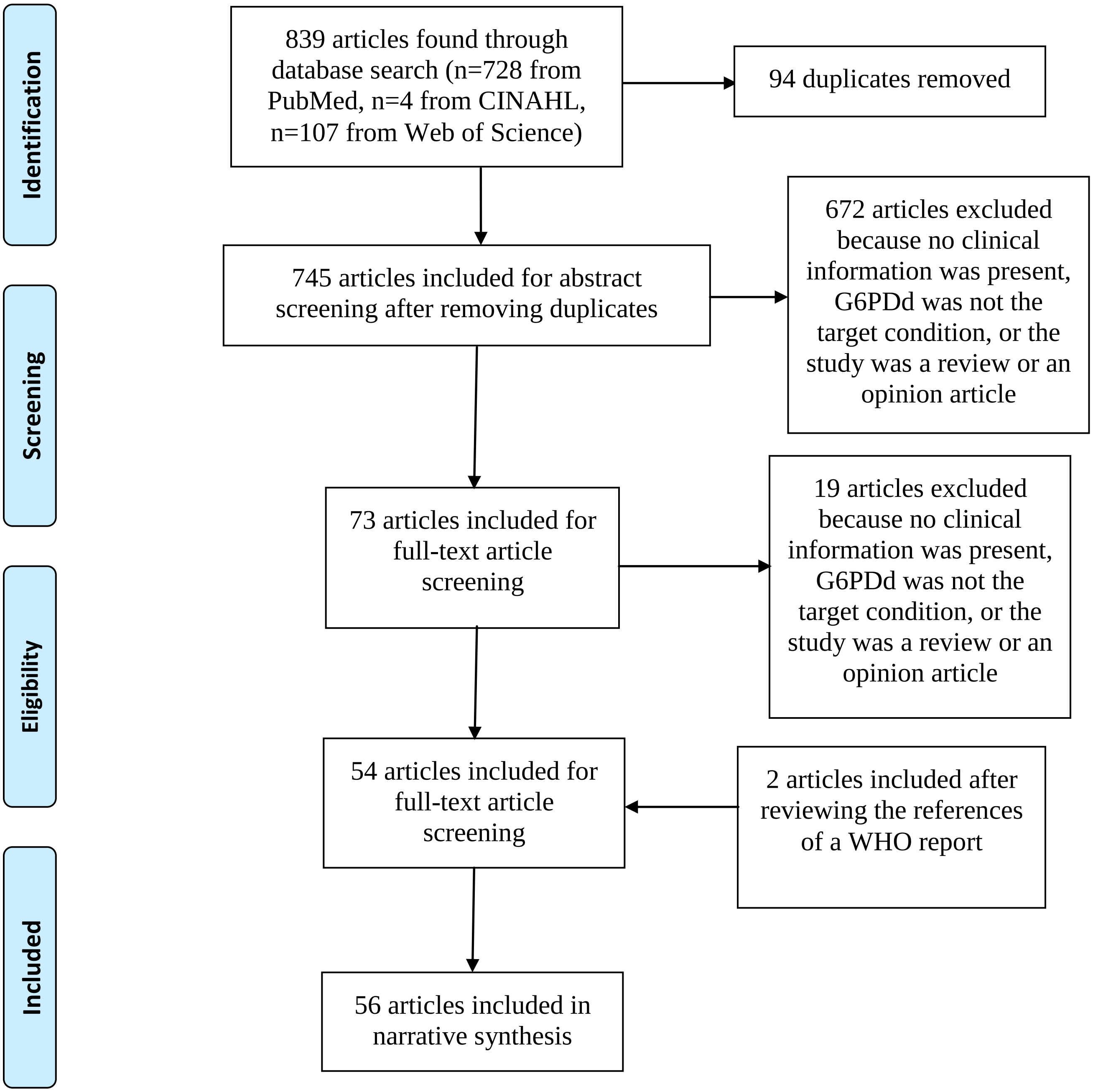

From: Moher D, Liberati A, Tetzlaff J, Altman DG, The PRISMA Group (2009). *P*referred *R*eporting *I*tems for *S*ystematic Reviews and *M*eta-*A*nalyses: The PRISMA Statement. PLoS Med 6(6): e1000097. doi:10.1371/journal.pmed1000097

For more information, visit www.prisma-statement.org.
