# Supplementary material for: Systematic review of the clinical manifestations of glucose-6-phosphate dehydrogenase deficiency in the Greater Mekong Subregion: implications for malaria elimination and beyond
Source: BMJ Glob Health. 2017 Aug 19;2(3):e000415. doi: 10.1136/bmjgh-2017-000415 (PMC5656182; doi:10.1136/bmjgh-2017-000415)
Supplement: Supplementary file 2 [file bmjgh-2017-000415supp002.pdf]

PROSPERO International prospective register of systematic reviews

G6PD deficiency in the Greater Mekong Subregion: a systematic review of the clinical manifestations and implications for malaria elimination

Ken Ing Cherng Ong, Hitomi Araki, Hodaka Kosugi, Moe Moe Thandar, Sophea Thoeun, Moritoshi Iwagami, Shigeyuki Kano, Masamine Jimba

Citation

Ken Ing Cherng Ong, Hitomi Araki, Hodaka Kosugi, Moe Moe Thandar, Sophea Thoeun, Moritoshi Iwagami, Shigeyuki Kano, Masamine Jimba. G6PD deficiency in the Greater Mekong Subregion: a systematic review of the clinical manifestations and implications for malaria elimination. PROSPERO 2016:CRD42016043146 Available from http://www.crd.york.ac.uk/PROSPERO_REBRANDING/display_record.asp?ID=CRD42016043146

Review question(s)

What are the major clinical manifestations and complications of Glucose-6-Phosphate Dehydrogenase deficiency (G6PDd) in the Greater Mekong Subregion?

Searches

We will search the following databases for published studies: PubMed/MEDLINE, CINAHL, and Web of Science. We will also hand search the references of relevant papers and WHO databases if necessary. We will include all published papers in the English language up till July 2016.

Our search strategy will combine both Medical Subject Headings (MeSH) terms and free text terms as follows:

(glucose-6-phosphate dehydrogenase deficiency OR G6PDd OR glucosephosphate dehydrogenase deficiency OR G-6-PD deficiency OR G6PD deficiency OR glucose-6-phosphate dehydrogenase OR G6PD OR G-6-PD) AND (Greater Mekong Subregion OR GMS OR Lao People's Democratic Republic OR Lao PDR OR Laos OR Thailand OR Vietnam OR Viet Nam OR Cambodia OR Myanmar OR Burma OR People's Republic of China OR Guangxi Zhuang Autonomous Region OR Yunnan Province OR Yunnan OR PRC)

Types of study to be included

Original research of all study designs will be included such as clinical trials, cohort studies, case-control studies, cross sectional studies, case series, and case reports as long as G6PD deficiency is the target condition, clinical information is available, and the population is from the Greater Mekong Subregion (Lao PDR, Thailand, Myanmar, Cambodia, Vietnam, Yunnan Province and Guangxi Zhuang Autonomous Region, People's Republic of China).

Condition or domain being studied

Glucose-6-phosphate dehydrogenase deficiency is the most common enzyme deficiency worldwide (Cappellini and Fiorelli, 2008). G6PD is an important enzyme that protects the red blood cells from oxidative stress through the production of nicotinamide adenine dinucleotide phosphate (NADPH) (Domingo et al., 2013). People who have this enzyme deficiency usually do not exhibit life threatening symptoms (Cappellini and Fiorelli, 2008). However, when exposed to triggers such as certain types of drugs, fava beans, and infections, acute hemolysis can occur (Cappellini and Fiorelli, 2008).

In the context of malaria, a G6PD deficient individual can develop severe hemolysis when given the antimalarial drug, primaquine (Monteiro et al., 2014b). However, primaquine is currently the only drug effective against the latent liver stage of vivax malaria (Kevin Baird, 2013). In addition, primaquine is also effective against the gametocyte stage of falciparum malaria (Matsuoka et al., 2007). Therefore, primaquine is indispensable in the current effort to eliminate malaria (World Health Organization, 2015).

The Greater Mekong Subregion (GMS) consists of Lao PDR, Thailand, Cambodia, Vietnam, Myanmar, and Yunnan Province and Guangxi Zhuang Autonomous Region in the People's Republic of China. The GMS encompasses an

area of 2.6 million km2 and sustains over 326 million lives (Asian Development Bank, 2016). Over the years, the GMS countries have made progress in reducing the number of malaria cases and also malaria deaths (World Health Organization, 2015). Despite the current progress in malaria elimination, the GMS countries are facing threats by the artemisinin resistant Plasmodium falciparum malaria parasite (Ashley et al., 2014). Therefore, to avoid malaria becoming untreatable with the current repertoire of drugs, it is essential that we aim for malaria elimination (World Health Organization, 2015).

Across malaria endemic countries, the G6PDd allele frequency is estimated to be about 8.0% (Howes et al., 2012). In the GMS countries, the hemolytic risk from G6PDd is high when both prevalence and variant severity are taken into consideration (Howes et al., 2012). In spite of this, no systematic review has been done on the major clinical manifestations and complications of G6PDd in the GMS countries. Therefore, this systematic review aims to compile evidence and identify the major clinical manifestations and complications of G6PDd in the GMS countries.

Participants/ population

Study population includes all those living in the GMS countries with G6PDd. We will exclude studies on migrant population from outside GMS residing in GMS countries.

Intervention(s), exposure(s)

Exposures are hemolysis-inducing drugs, fava beans, and hemolysis-inducing infections.

Comparator(s)/ control

We will also try to include information on the population living in the GMS countries without G6PDd but exposed to hemolysis-inducing drugs, fava beans, or hemolysis-inducing infections.

Outcome(s)

Primary outcomes

Acute hemolytic anemia, drug-induced hemolysis, favism, infection-induced hemolysis, neonatal jaundice, chronic non-spherocytic anemia.

Secondary outcomes

None

Data extraction, (selection and coding)

At least two researchers will be involved in the process from literature search, article screening and data extraction. In the event of conflict, a third researcher will be consulted.

Risk of bias (quality) assessment

Risk of bias assessment will not be carried out.

Strategy for data synthesis

Narrative synthesis.

Analysis of subgroups or subsets

None.

Dissemination plans

Publication in a peer-reviewed scientific journal.

Contact details for further information

Mr Ong

Department of Community and Global Health,

The University of Tokyo, 7-3-1 Hongo Bunkyo-ku,

Tokyo 113-0033 JAPAN.

kenicong@m.u-tokyo.ac.jp

Organisational affiliation of the review
None

Review team
Mr Ken Ing Cherng Ong, Department of Community and Global Health, Graduate School of Medicine, The University of Tokyo, Tokyo, Japan
Ms Hitomi Araki, Department of Community and Global Health, Graduate School of Medicine, The University of Tokyo, Tokyo, Japan
Mr Hodaka Kosugi, Department of Community and Global Health, Graduate School of Medicine, The University of Tokyo, Tokyo, Japan
Dr Moe Moe Thandar, Department of Community and Global Health, Graduate School of Medicine, The University of Tokyo, Tokyo, Japan
Mr Sophea Thoeun, Department of Community and Global Health, Graduate School of Medicine, The University of Tokyo, Tokyo, Japan
Dr Moritoshi Iwagami, Department of Tropical Medicine and Malaria, Research Institute, National Center for Global Health and Medicine, Tokyo, Japan and JICA/AMED SATREPS project Lao-Japan Parasitology Laboratory Institut Pasteur du Laos, Vientiane, Lao PDR
Dr Shigeyuki Kano, Department of Tropical Medicine and Malaria, Research Institute, National Center for Global Health and Medicine, Tokyo, Japan
Dr Masamine Jimba, Department of Community and Global Health, Graduate School of Medicine, The University of Tokyo, Tokyo, Japan

Anticipated or actual start date
01 August 2016

Anticipated completion date
31 January 2017

Funding sources/sponsors
This study is a part of the ‘Development of innovative research technique in genetic epidemiology of malaria and other parasitic diseases in Lao PDR for containment of their expanding endemicity’ project under the Science and Technology Research Partnership for Sustainable Development (SATREPS) supported by Japan International Cooperation Agency (JICA) and Japan Agency for Medical Research and Development (AMED).

Conflicts of interest
None known

Language
English

Country
Japan, Laos

Subject index terms status
Subject indexing assigned by CRD

Subject index terms
Antimalarials; Glucosephosphate Dehydrogenase Deficiency; Humans; Immunologic Tests; Malaria

Stage of review
Ongoing

Date of registration in PROSPERO
19 July 2016

Date of publication of this revision
17 January 2017

Stage of review at time of this submission	Started	Completed
Preliminary searches	No	No
Piloting of the study selection process	No	No
Formal screening of search results against eligibility criteria	No	No
Data extraction	No	No
Risk of bias (quality) assessment	No	No
Data analysis	No	No

PROSPERO
International prospective register of systematic reviews
The information in this record has been provided by the named contact for this review. CRD has accepted this information in good faith and registered the review in PROSPERO. CRD bears no responsibility or liability for the content of this registration record, any associated files or external websites.
