# Supplementary material for: Systematic review of the clinical manifestations of glucose-6-phosphate dehydrogenase deficiency in the Greater Mekong Subregion: implications for malaria elimination and beyond
Source: BMJ Glob Health. 2017 Aug 19;2(3):e000415. doi: 10.1136/bmjgh-2017-000415 (PMC5656182; doi:10.1136/bmjgh-2017-000415)
Supplement: Supplementary file 4 [file bmjgh-2017-000415supp004.pdf]

S1 Table. Summary of included studies.

No.	Reference	Type of study	Country (City)	Year	Population (Age, gender, if available)	Population size	Diagnostic methods	Exposures (Dosage, if available)	Clinical outcome	Remarks
1	Areekul 1996 [24]	Case report	Thailand (Bangkok)	1996	13, 14, 29 years old G6PD deficient male patients with typhoid fever	3	N/A	Typhoid fever	Hemolysis, hemoglobinuria	This paper described the elevated serum transcobalamin II levels in three G6PD deficient patients with typhoid fever
2	Arnolda 2015 [49]	Retrospective cohort	Myanmar (Yangon and Mandalay)	2015	Neonates treated on new phototherapy machines over 13-20 months at two pediatric referral hospitals in Myanmar.	590	Methemoglobin reduction test	N/A	Acute bilirubin encephalopathy (ABE)	G6PD deficiency was a risk factor for ABE (only in one hospital).
3	Bancone 2016 [57]	Repeated cross-sectional and cohort study	Thai-Myanmar border	2016	Male and female villagers older than six months	819	Fluorescent spot test	Primaquine (single dose 0.25 mg base/kg)	Dizziness, fractional hemoglobin fall among G6PD deficient villagers, itchiness	Authors concluded that a single low dose of primaquine (0.25mg/kg) can be used safely without prior G6PD testing
4	Na Bangchang 1994 [18]	Cohort study	Thailand (Bangkok)	1994	13 G6PD normal and 13 G6PD deficient Thai male vivax malaria patients	26	N/A	Primaquine (daily dose of 15mg for 14 days)	Hemolysis was observed in all G6PD deficient patients	G6PD status is a useful predictor for the risk of hemolysis after treatment with primaquine
5	Buchachart 2001[58]	Prospective cohort study	Thailand (Bangkok)	2001	Vivax malaria patients	364	Fluorescent spot test	Primaquine (daily dose of 15mg for 14 days)	Significantly decreased hematocrit level among G6PD deficient individuals	Daily doses of 15 mg of primaquine for 14 days following a full course of chloroquine when prescribed to Thai G6PD patients where the Mahidol variant is predominant are relatively safe
6	Burgoine 2010 [19]	Case report	Thai-Myanmar border	2010	35-year-old male vivax malaria patient	1	Fluorescent spot test, genotyping	Primaquine (overdosing)	Intravascular hemolysis	In *P.vivax* endemic areas, patients should be tested for G6PD deficiency and sufficiently informed of the side effects of primaquine before administration
7	Butthep 2000 [50]	Cross-sectional	Thailand (Bangkok)	2000	28 anemic patients (15 with iron deficiency anemia, and 13 with hemolytic anemia including 9 G6PD deficient and 4 G6PD deficient combined with HbE trait or alpha thalassemia trait); 25 normal patients	53	G6PD assay	N/A	Increase in mean corpuscular reticulocyte volume in anemic G6PD deficient patients	
8	Chanda 2015 [69]	Cohort study	Thailand (Bangkok)	2015	18-24-year-old female volunteers	36	Genetic characterization	Strenuous exercise	An increase in total microparticles (TTMPs) concentration was significantly higher in individuals with G6PD Viangchan as compared to G6PDnormal.	Individuals with G6PD Viangchan variant may participate in moderate-intensity exercise without higher oxidative stress compared to G6PD normal individuals
9	Charoenlarp 1972 [17]	Cohort study	Thailand (Bangkok)	1972	18-26-year-old Thai males with G6PD deficiency	7	NADPH quantification	Primaquine (single dose of 45mg)	Hemolysis	
10	Charoenlarp 1973 [20]	Cohort study	Thailand (Bangkok)	1973	Healthy Thai male with G6PD deficiency	3	NADPH quantification	Primaquine (two 14-day daily dose of 15mg)	Hemolysis	The 15 mg dose of primaquine in Thais caused hemolysis as rigourously as that induced by 30 mg in the African type
11	Chin 1973 [59]	Non-randomized controlled study	Thailand (Trad)	1973	Adult males 15 years of age or older with a positive blood smear for falciparum malaria	88	Fluorescent spot test	Sulfalene (1g)-trimethoprim (0.5g) , Sulphormethoxine (1g)-pyrimethamine (0.05g)	No significant toxic side reactions	

12	Chongsuphajaisiddhi 1987 [60]	Case series	Thailand (Bangkok)	1987	5-12 years old children with acute, uncomplicated falciparum malaria	84	N/A	Mefloquine (a single dose of 18-29mg base per kg body weight)	No significant toxic side reactions except vomiting, diarrhea, and mild abdominal pain	Mefloquine was effective in treating falciparum malaria
13	Chuansumrit 2012 [25]	Case series	Thailand (Bangkok)	2012	Hematologic-oncologic pediatric patients with dengue infection	907	N/A	Dengue infection	Anemia from acute hemolysis and hemoglobinuria was prominent among patients with G6PD deficiency	
14	Devakul 1969 [26]	Case series	Thailand (Bangkok)	1969	Thai male infected with malaria who had been transferred from a mental hospital	10	N/A	Falciparum malaria	Hemolysis	Considerable total hemoglobin concentration fall in G6PD deficient patients
15	Everett 1977 [21]	Cohort study	Cambodia	1977	Khmer Air Force male troops	106	N/A	Primaquine (daily dose of 15mg for 14 days)	Primaquine induced significant, but not dangerous hemolysis in G6PD deficient individuals	Primaquine induced a significant but safe level of hemolysis in subjects with G6PD Mahidol
16	Flatz 1963 [51]	Cross-sectional	Thailand (Nakorn Rajasima, Surin, Bangkok)	1963	122 married women over the age of 36 years from North-east Thailand (Nakorn Rajsima and Surin), 1327 cord blood samples from newborns in Bangkok, 11patients thought to suffer from cerebral palsy due to bilirubin encephalopathy (8 males, 3 females) and 22 children with similar age and sex distribution as a control group	1482	Methemoglobin reduction test	Oxidative drugs/chemicals	G6PD deficient group had more severe, unexplained jaundice	G6PD deficiency is a risk factor for cerebral palsy due to bilirubin encephalopathy. Neonatal hyperbilirubinemia with bilirubin encephalopathy seemed to be the main negative balancing factor for the G6PD polymorphism in Thailand
17	Flatz 1964 [38]	Cross-sectional	Thailand (Chiang Mai)	1964	Newborn infants	236	Dye reduction test, methemoglobin reduction test	N/A	Jaundice	The risk of G6PD deficient newborn infants developing severe jaundice in the absence of drug administration seems to be low under the conditions in Northern Thailand
18	Harinasuta 1983 [61]	Randomized controlled trial	Thailand (Bangkok)	1983	Male patients suffering from acute, uncomplicated, falciparum malaria	147	Quantitative test	Single dose of either 500, 750, or 1000mg of mefloquine	Nausea, vomitting, diarrhea	Mefloquine was well tolerated, no evidence of hemolysis
19	Nguyen 2009 [52]	Cross-sectional	Vietnam (Ho Chi Minh City, Binh Phuoc)	2009	General population and hemoglobinuria patients	606	Methylene blue reduction test, G-6-PDH kit, micromethemoglobin reduction test	Active malaria, ingestion of oxidant drugs such as primaquine	Hemoglobinuria	G6PD deficiency is strongly associated with hemoglobinuria
20	Kahn 1978 [70]	Case report	France (patient was from Laos)	1978	28 years old male patient admitted to the hospital with symptomatology of paroxysmal nocturnal hemoglobinuria (PNH).	1	G6PD assay	N/A	Hemoglobinuria	A new G6PD variant called G6PD Vientiane was discovered.
21	Karwacki 1989 [22]	Case report	Thailand (Chanthaburi)	1989	28 years old Royal Thai Marine Militia Soldier	1	Methemoglobin reduction test	Primaquine (15mg daily for 3 days)	Vomitting, hemolysis, acute renal failure	The unusal nature of the case reported here could mean either underreporting of adverse events due to G6PD deficiency or the rarity of severe G6PD induced hemolysis in Thailand

22	Kheng 2015 [23]	Clinical trial	Cambodia (Pailin, Anlong Venh, Oddar Meanchey, Veal Veng, and Pursat)	2015	Acute vivax malaria patients	75	Quantitative G6PD assay (Trinity Biotech), genotyping	Primaquine (0.75mg/kg for 8 weeks)	Significant but transient fall in hemoglobin level, and one patient received a blood transfusion	Weekly primaquine in G6PDd patients mandates medical supervision and pre-treatment screening for G6PD status.
23	Khim 2013 [42]	Cross-sectional	Cambodia (mainly western Cambodia)	2013	Malaria infected patients from 19 health centres in Cambodia	2408	G6PD quantitative assay (Trinity Biotech)	Malaria infection	Anemia is more frequent in G6PD deficient patients	Severe G6PDd is prevalent mostly in western Cambodia where artemisinin resistance is also present. Therefore primaquine safety and G6PD point of care evaluation studies are needed
24	Kitayaporn 1991[37]	Cross-sectional	Thailand (Chanthaburi)	1991	Patients aged 16-60 years	395	Fluorescent spot test	Fava bean consumption	No evidence of hemolysis resulting from G6PD deficiency	
25	Kotepui 2016 [43]	Cross-sectional	Thailand (Tak)	2016	Malaria patients	245	Fluorescent spot test	Malaria infection	G6PD deficient patients have higher monocyte counts.	G6PD deficient RBCs have no protection against P. falciparum infection.
26	Krudsood 2006 [62]	Randomized, open-label, prospective study	Thailand (Bangkok)	2006	16-51 years old vivax malaria patients	141	Screening test	Primaquine 30mg daily for 7 days, elubaquine 25mg once daily for 7 days	Mean hematocrit was significantly reduced in patients treated with primaquine compared with elubaquine	Elubaqine might have less oxidative toxicity compared to primaquine
27	Lampe 1975 [44]	Cross-sectional	Thailand (Bangkok)	1975	3 groups of patients seen at Children's Hospital, Bangkok (typhoid fever, outpatients who were free of bacterial disease, patients with serious bacterial disease)	231	Methemoglobin-elution test	Typhoid fever, bacterial diseases such as meningitis	Increased susceptibility to infection	G6PD deficiency is associated with typhoid fever and susceptibility to certain infectious diseases
28	Laosombat 2006 [36]	Retrospective descriptive	Thailand (Songkla)	2006	G6PD deficient children (210 boys, 15 girls)	225	G6PD assay	Ingestion of fava beans, drugs, infections	Favism, anemia, hemoglobinuria, jaundice	Favism was found in 3.6% of G6PDd Thai children
29	Laosombat 2005 [32]	Retrospective descriptive	Thailand (Songkla)	2005	G6PD deficient children (210 boys, 16 girls)	225	G6PD Assay	Ingestion of fava beans, drugs, infections	Neonatal jaundice, acute hemolysis, and drug-induced acute hemolysis were observed in these patients.	A new variant of G6PD gene was describe: g6PD Songklanagarind
30	Lederer 1988 [45]	Cross-sectional	Thailand	1988	Adult male malaria patients (either falciparum or vivax)	192	Fluorescent spot test	Malaria infection	G6PD deficient patients had significantly less gastrointestinal disturbances, higher serum glutamic oxalacetic transaminase, and significantly lower blood urea nitrogen compared with the G6PD normal malaria cases	G6PD deficiency has no significant influence on the clinical presentation of malaria.
31	Myat 1994 [63]	Clinical trial	Myanmar (Lashio, Mandalay)	1994	Malaria patients	63	Methemoglobin reduction test	600 mg quinine 3 times per day for 7 days followed by 45mg single dose primaquine for gametocytes (falciparum malaria) and 45mg weekly primaquine for 8 weeks for vivax malaria	No hemolysis was reported and no marked change in methemoglobin concentration was observed	Toxicity of primaquine might be dose related
32	Noedl 2002 [46]	Cross-sectional	Thailand (Bangkok)	2002	Microscopically confirmed falciparum malaria patients of both genders	119	Fluorescent spot test	Malaria infection	G6PD deficient patients have shorter median fever clearance time compared to G6PD normal individuals	

33	Oo 1995 [47]	Cross-sectional	Myanmar (Yangon)	1995	Bamar male aged 19-45 with severe falciparum malaria	383	Methemoglobin reduction test	Malaria infection	The mean parasitemia level in patients with severe G6PDd variant was significantly lower compared to normal and mild deficiency variants	The double genetic defect of thalassemia trait and severe G6PD deficiency appeared to confer some degree of protection against malaria.
34	Panich 1973 [33]	Case report	Thailand	1973	Hospital patients	4	Methemoglobin reduction test	Drug, and/or infection	Acute hemolysis, hemoglobinuria	G6PD Union (Thai) resulted in acute hemolysis
35	Panich 1970 [48]	Case report	Thailand	1970	Hospital patients	8	Rapid screening dye test.	Infections	Anemia, jaundice, hemoglobinuria	Hereditary elliptocytosis is associated with G6PD deficiency
36	Phornphutkul 1969 [39]	Case series	Thailand	1969	Newborn infants	1785	Methemoglobin reduction test	N/A	Neonatal jaundice	G6PD deficiency might be an important cause of severe early neonatal jaundice
37	Pornprasert 2013 [68]	Case series	Thailand (Chiang Mai)	2013	People living with HIV	109	Fluorescent spot test, genotyping	Highly active antiretroviral therapy	Non reported	HAART did not cause hemolytic anemia and hyperbilirubinemia in people living with HIV
38	Pornprasert 2013 [53]	Cross-sectional	Thailand (Chiang Mai)	2013	Thalassemia patients	410	Fluorescent spot test	N/A	Non reported	In the absence of a high oxidative stress condition, G6PD deficiency did not enhance red blood cell pathology or induce more anemic severity in thalassemia patients
39	Pornprasert 2016 [71]	Cross-sectional	Thailand (Chiang Mai)	2016	Volunteer students of one school in a fluoride endemic area, and two schools in control areas	481	Fluorescent spot test	Fluoride exposure	Students in fluoride endemic area with hematological disorders (iron deficiency, thalasemia, G6PDd) had the lowest levels of Hb, MCH, MCHC, and the highest platelet counts	Fluoride might exacerbate the effect of G6PD deficiency .
40	Poshyachinda 1978 [34]	Controlled trial	Thailand (Bangkok)	1978	G6PD normal and G6PD deficient volunteers	34	Methemoglobin reduction test	4, 4' Diformyl diaminodiphenylsulfone (DFD) (single dose of either 400, 800, 1200, and 1600 mg)	Hemolysis	G6PDd inividuals are susceptible to DFD-induced hemolysis.
41	Prachukthum 2009 [54]	Case-control	Thailand (Bangkok)	2009	Hyperbilirubinemic neonates and normal neonates	177	G6PD assay	Blood group incompatibility, and variants of gene *UGT1A1* at nt 211	Hyperbilirubinemia	G6PD deficiency is associated with hyperbilirubinemia
42	Sanpavat 2005 [72]	Retrospective descriptive	Thailand (Bangkok)	2005	Neonates who underwent exchange transfusion	165	N/A	Blood group incompatibility	Hyperbilirubinemia	G6PD deficiency caused hyperbilirubinemia.
43	Shanks 1992 [64]	Randomized controlled trial	Thailand (Thai-Cambodian border)	1992	Royal Thai Marine Militia on guard duty along the Thai-Cambodian border	438	N/A	Doxycycline (100mg/day)	No clinical outcome reported for G6PD deficient individuals	Authors concluded that doxycycline is an effective chemoprophylactic drug for malaria
44	Sicard 1978 [35]	Case report	Lao PDR	1978	Soldiers	50	Electrophoresis	Chloroquine (single dose of 600mg)	Severe hemolytic anemia, acute renal failure, hemoglobinuria	
45	Silachamroon 2003 [65]	Randomized controlled trial	Thailand (Bangkok)	2003	Vivax malaria patients	801	Screening test	Primaquine (0.6 mg/kg for 14)	Decreased hematocrit level	In Thailand where the prevalent variants of G6PD deficiency are mild, G6PDd patients will be unable to tolerate high dose primaquine
46	Tachavanich 2009 [55]	Cross-sectional	Thailand	2009	Homozygous hemoglobin E individuals	76	N/A	N/A	Iron deficiency anemia	G6PD deficiency had no additional adverse effect on hematological parameters of homozygous hemoglobin E individuals
47	Talalak 1969 [41]	Case report	Thailand	1969	Thai boy	1	G6PD assay	N/A	Congenital nonspherocytic hemolytic anemia, jaundice	This new variant found in the Thai boy was named G6PD Bangkok

48	Tanphaichitr 2002 [27]	Case series	Thailand (Bangkok)	2002	Males aged 1-13 years diagnosed with dengue haemorrhagic fever (DHF)	89	G6PD assay	Dengue hemorrhagic fever	Gastrointestinal bleeding, hemolysis, shock, renal and liver failure	Patients with G6PDd suffer from DHF more than patients with normal G6PD activity
49	Tanphaichitr 2011 [28]	Case report	Thailand (Bangkok)	2011	Two families	7	G6PD assay	Dengue hemorrhagic fever	Neonatal jaundice, acute hemolysis, chronic hemolysis.	A novel G6PD variant, G6PD Bangkok Noi was described
50	Tanphaichitr 1995 [40]	Cross-sectional	Thailand (Bangkok)	1995	Newborn male infants	505	G6PD assay	Infections, blood group incompatibility	Neonatal jaundice	G6PDd male infants are more susceptible to develop neonatal jaundice compared to G6PD normal male infants
51	Tanphaichitr 1982 [29]	Case series	Thailand (Bangkok)	1982	Children with typhoid fever	79	G6PD quantitative assay, methemoglobin reduction test	Typhoid fever	Acute, intravascular hemolysis among G6PD deficient patients, and transient, acquired G6PD deficiency among normal individuals	Typhoid fever can cause transient, acquired low G6PD level in G6PD normal subjects due to bone marrow suppression
52	Thisyakorn 1987 [31]	Case series	Thailand (Bangkok)	1987	Children with enteric fever.	192	Methemoglobin reduction test	Typhoid fever	Acute hemolytic anemia.	G6PDd children treated with trimethoprim-sulfamethoxazole might be at risk of hemolysis
53	Tran 1996 [56]	Prospective descriptive study	(Vietnam) Ho Chi Minh City	1996	Patients with blackwater fever	50	Methemoglobin reduction test	Quinine ingestion, malaria infection.	Blackwater fever (Hemoglobinuria) is associated with G6PD deficiency	
54	Wanachiwanawin 1990 [30]	Case report (Retrospective)	Thailand (Bangkok)	1990	G6PD deficient patients with viral hepatitis	9	Rapid dye reduction test, methemoglobin reduction test	Viral hepatitis.	Acute hemolysis, severe hyperbilirubinemia, renal failure.	
55	Song 2010 [66]	Case series	Cambodia (Kampong Speu, Kampot)	2010	All villagers in the targeted villages	6040	G-6-PDH kit (Trinity Biotech)	Primaquine (9mg for adults)	No severe adverse events reported	Mass drug administration of artemisinin-piperaquine and low doses of primaquine can be an effective strategy for eliminating malaria
56	Takeuchi 2010 [67]	Randomized controlled trial	Thailand (Ratchaburi)	2010	Patients 3 years and above with microscopically-confirmed *P.vivax* infection	216	Genotyping	Primaquine (5mg for 14 days)	None reported	Directly-observed therapy (DOT) for primaquine helped to improve adherence
